# The CrowdWater game: A playful way to improve the accuracy of crowdsourced water level class data

**DOI:** 10.1371/journal.pone.0222579

**Published:** 2019-09-26

**Authors:** Barbara Strobl, Simon Etter, Ilja van Meerveld, Jan Seibert

**Affiliations:** 1 Department of Geography, University of Zurich, Zurich, Switzerland; 2 Department of Aquatic Sciences and Assessment, Swedish University of Agricultural Sciences, Uppsala, Sweden; Torrens University Australia, AUSTRALIA

## Abstract

Data quality control is important for any data collection program, especially in citizen science projects, where it is more likely that errors occur due to the human factor. Ideally, data quality control in citizen science projects is also crowdsourced so that it can handle large amounts of data. Here we present the CrowdWater game as a gamified method to check crowdsourced water level class data that are submitted by citizen scientists through the CrowdWater app. The app uses a virtual staff gauge approach, which means that a digital scale is added to the first picture taken at a site and this scale is used for water level class observations at different times. In the game, participants classify water levels based on the comparison of the new picture with the picture containing the virtual staff gauge. By March 2019, 153 people had played the CrowdWater game and 841 pictures were classified. The average water level for the game votes for the classified pictures was compared to the water level class submitted through the app to determine whether the game can improve the quality of the data submitted through the app. For about 70% of the classified pictures, the water level class was the same for the CrowdWater app and game. For a quarter of the classified pictures, there was disagreement between the value submitted through the app and the average game vote. Expert judgement suggests that for three quarters of these cases, the game based average value was correct. The initial results indicate that the CrowdWater game helps to identify erroneous water level class observations from the CrowdWater app and provides a useful approach for crowdsourced data quality control. This study thus demonstrates the potential of gamified approaches for data quality control in citizen science projects.

## 1. Introduction

Data quality and quality control are frequently discussed for citizen science projects because these data are generally perceived to be less accurate than traditional data due to human errors. Nonetheless several studies have shown that citizen science data can be as accurate as data from experts [1–3]. Data quality control in citizen science has several purposes, the most obvious being the improvement of the data quality. Additionally, improved data quality also increases the credibility of the data for the users and the confidence of the citizen scientists in their ability to submit useful data [4,5].

Different data quality control approaches have been developed for citizen science projects within different scientific fields and for different data collection approaches [4,6–8]. Wiggins et al. [8] summarised 18 approaches for data quality control, which can be grouped into approaches before, during and after data collection. These include training participants and providing tutorial materials [9,10], filtering of the incoming data based on the plausibility of the data and the likelihood for a particular geographic region [6,10–14], bias correction, for example for presence only data [10,11,15,16], and review of incoming data [4,8,11,17,18].

The review approach includes reviews by professional scientists, reviews by experienced contributors or regional experts, and peer-reviews by multiple parties [8]. The three review approaches can also be combined within a project, e.g. by asking the public to flag certain entries (peer-review), which are then reviewed by experts [6,18]. Review through professionals is a time-consuming task and can only be done in citizen science projects with a limited amount of incoming data [4]. While all non-fully automated data quality control approaches might be time-consuming, the effort becomes more doable if it can be shared by many people. Review by experienced contributors or regional experts can therefore also be used for large projects. However, it opens the question whom to assign this *“ambassador-status”*, and depending on the data volume and number of ambassadors it might still be a lot of work for a few dedicated volunteers [11,18]. Peer-review by multiple parties is a method to crowdsource data quality control. The quality of the review is insured through multiple assessments so that individual mistakes or misclassifications are insignificant when all assessments are taken into account. Through peer review, the quality control mechanism is scalable, so that it can even be used for big projects. Furthermore, it ensures that citizen scientists are involved in both data collection and data quality control.

There are many examples of peer-review in the field of citizen science, such as projects related to Volunteered Geographic Information [7,19], such as OpenStreetMaps [20], in projects where volunteers make visual comparisons of spatial patterns, such as Pattern Perception [21], Cyclone center [22] and Galaxy Zoo [23], and in projects where volunteers assess pictures, such as Snapshot Serengeti [24] and Cropland Capture [17]. As with many citizen science projects and tasks, a major difficulty associated with the peer-review approach is the recruitment and retention of a sufficient number of reviewers [17,21–25]. Depending on the project, the scientific field and the specific task at hand, different strategies can be employed. A frequently applied strategy, especially for online citizen science projects, is the gamification of tasks [26–28].

Gamification can range from simple points and leaderboards to more immersive games with complex storylines [28]. Different phrases are used in the literature for these types of games, such as citizen science games [29,30], knowledge games [31], games with a purpose [32,33] or serious games [29]. Examples of projects that gamified their interaction with citizen scientists are Foldit, StallCatchers, Phylo, Serengeti Pictures and Cropland Capture. A comprehensive list of gamified citizen science projects can be found on www.citizensciencegames.com.

Many of these games were successful in finding a large number of participants: > 2 500 players in Cropland Capture [34], > 12 000 players in Phylo [35], and > 57 000 players in Foldit [36]. The different topics of the games make comparisons between them difficult, but most publications describe the games as a success. The project Foldit mentions that their players can *“produce structure solutions of the highest quality”* [37] and Curtis [38] says that *“the games [Foldit*, *Phylo and EteRNA] have the potential to greatly improve our understanding of the genetic processes underlying important diseases”*. For Cropland Capture, the conclusions were slightly mixed *“At first glance […] volunteers are highly effective at rating photographs and satellite imagery for the presence of cropland*.*”*, but *“extracting a reliable signal from crowdsourced data without guidance from expert validations is not possible for this type of task*.*”* [34].

**Fig 1:**
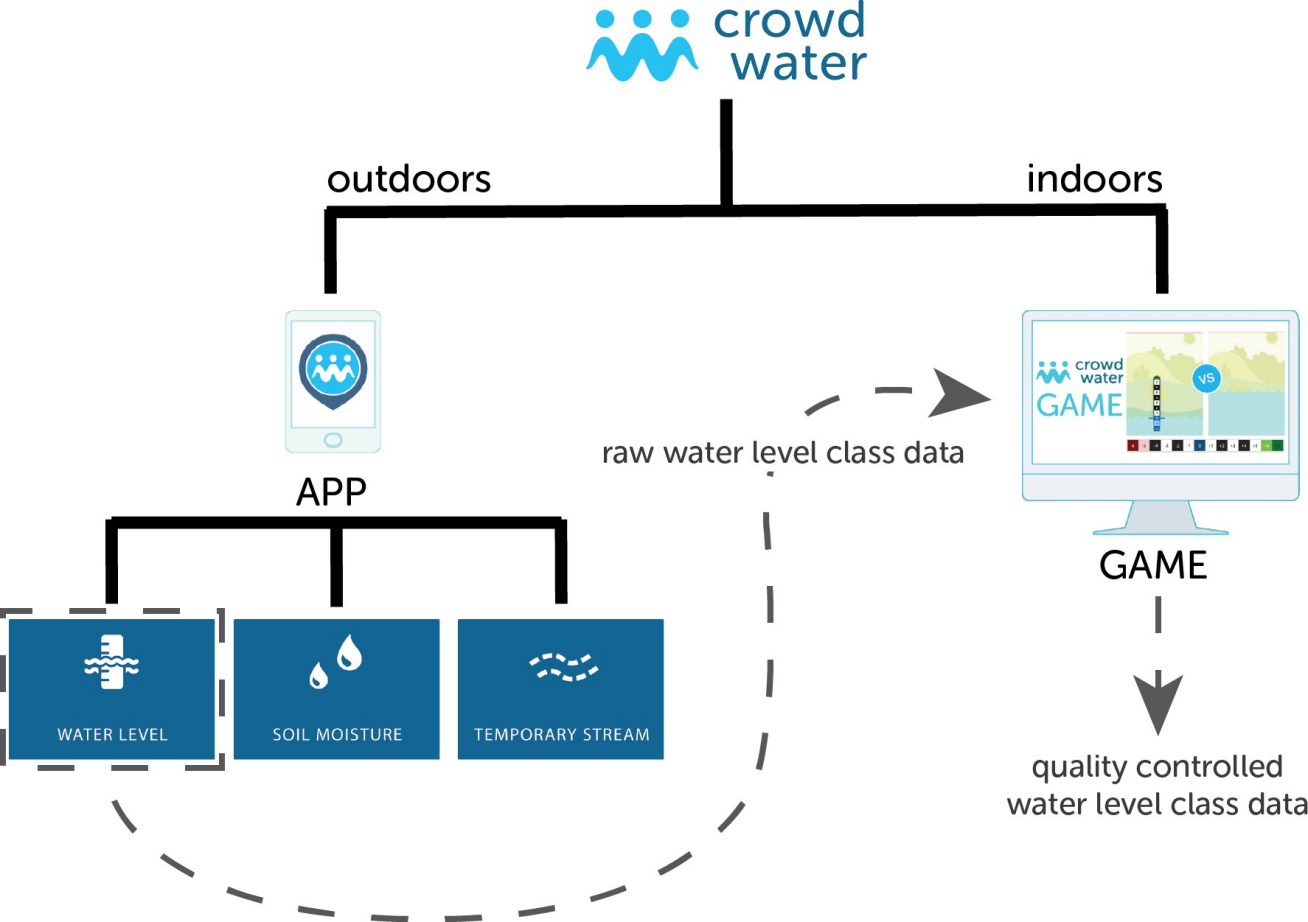
Schematic overview of the CrowdWater project, showing the connection between the CrowdWater app and game.

This paper focuses on the value of the online CrowdWater game (https://crowdwater.ch/en/crowdwater-game/) to check and improve the accuracy of crowdsourced water level class data submitted via the CrowdWater app. The CrowdWater game can be described as a citizen science game for which the primary purpose is data quality control rather than education. One of the main differences between the CrowdWater game and most other citizen science games is the complementarity of the tasks (collection and correction) in the CrowdWater project. The CrowdWater project asks citizens to submit water level class data for streams and rivers through an app [39], which in turn are checked by (other) participants through the online CrowdWater game. Therefore, unlike games such as Foldit, Phylo or Cropland Capture, the CrowdWater game does not produce data, but checks the quality of the crowdsourced data. This means that there are two potential entry points into the project (the app and the game; Fig 1) and that there is a range of tasks and interactions available for participants. This might help to *“sustain engagement over time”* [27]. iNaturalist [[40] and iSpot [41] have a similar complementarity as the CrowdWater project, by asking the citizen scientists to collect pictures of plants and animals and by also asking citizen scientists with good species recognition abilities to help classify these pictures.

The specific research questions for this study were:

1. Can the CrowdWater game be used to correct mistakes in the data submitted through the CrowdWater app?
2. Can players correctly identify unsuitable observations through the report function in the game?
3. Is the assignment of the water level class by regular players more accurate than for novice players?
4. What motivates participants to play the CrowdWater game?

## 2. Methods

### 2.1 The CrowdWater app

The CrowdWater app [39,42] can be used by citizen scientists worldwide to collect hydrological data. Currently, the app can be used to collect data for three parameters: water level class (and as an advanced option, streamflow), soil moisture, and the occurrence of flow in temporary streams. The CrowdWater game was developed as a data quality control mechanism for the water level class data. To report changes in water levels with the app, users first have to create a reference picture, which is a picture of a stream with a virtual staff gauge that is inserted digitally onto the picture like a sticker. Hence the staff gauge only exists in the reference picture and no physical installations are needed. The size of the virtual staff gauge is controlled by the user but the number of classes is fixed at ten. The virtual staff gauge is placed in the picture in such a way that per definition the water level in the reference picture is always at class zero. At a later time, the user who has made the reference picture or any other citizen scientist visiting the same location can look at the initial reference picture and compare the water level in the current situation with that on the reference picture. They select the water level class that they think best represents the current situation and upload a picture of the new situation. This is called *“observation”* and results in a time series of water level class data with relative values of the water level for that particular location, rather than time series of actual (metric) water level values. For further information on the CrowdWater app, as well as the use of the virtual staff gauge approach, we refer the reader to the following publications [39,43–45].

### 2.2 The CrowdWater game

#### 2.2.1 The game

The CrowdWater game is a casual game, which means that very little time, background knowledge, experience or training is needed to start playing the game [46]. The instruction manual is available on the homepage of the CrowdWater project and can be read easily in 5 to 10 minutes. In addition, there is a short (<2 min) movie explaining the game (https://crowdwater.ch/en/instructions/). The task for the players can be described as a series of “*microtasks”*, which refers to “*[…] systems [that] achieve high quality*, *typically as good as or better than expert annotators*, *through extensive use of redundancy and aggregation*.” [47]. The gamification aspects of the CrowdWater game include championships, rounds, points and leaderboards.

**Fig 2:**
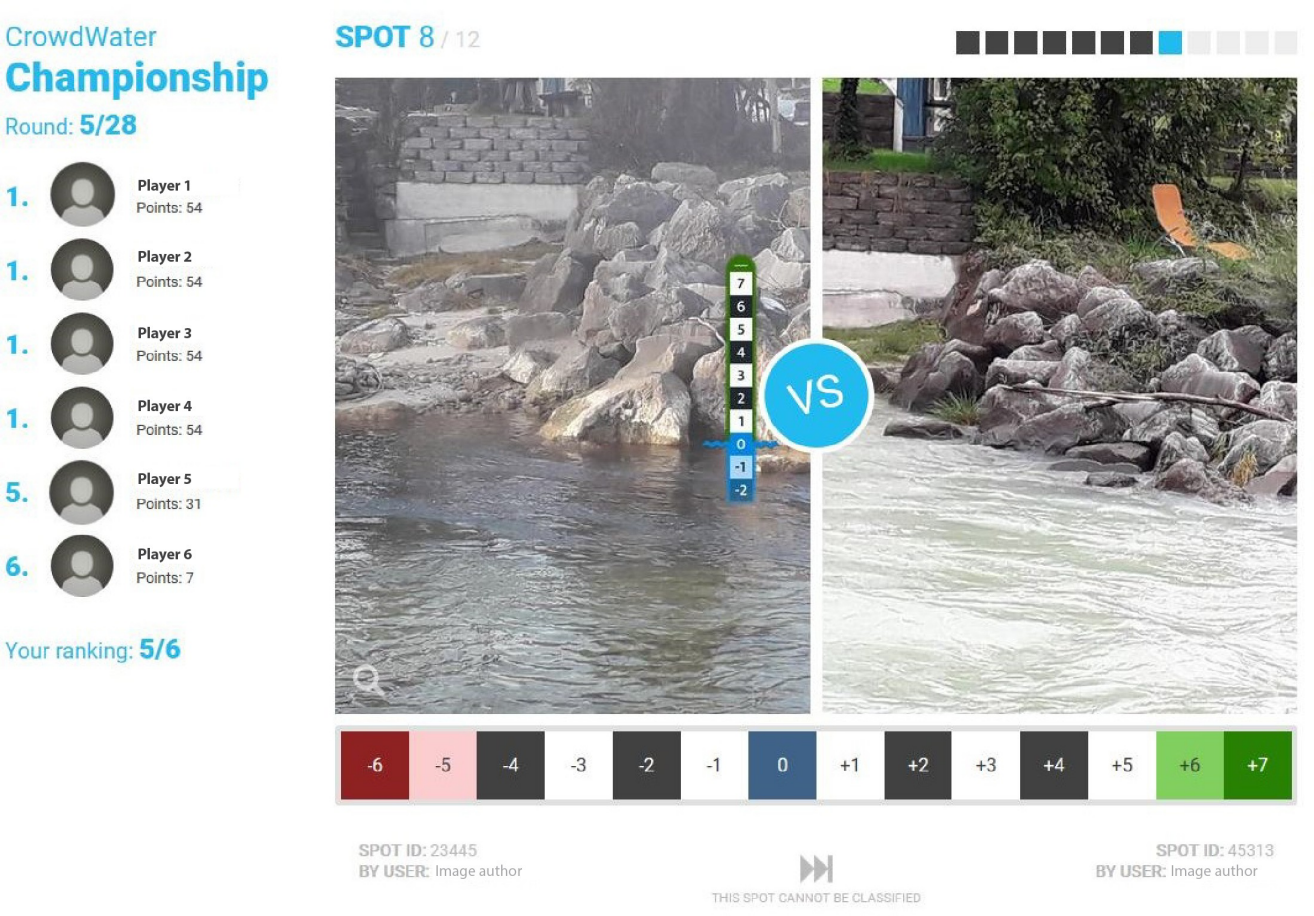
Screenshot of the CrowdWater game. The left picture shows the reference picture with the virtual staff gauge. The right picture shows an observation for the same spot at a different time. The player has to estimate the water level class for the picture on the right by comparing the water level and features in the stream or on the stream bank for both pictures. On the left hand side the scores of the top ten players for this round of the game are shown. The report button at the bottom can be used for pictures that cannot be classified. The squares at the top indicate the number of comparisons completed in this round of the game so far (black) and comparisons still to come (grey).

The crowdsourced water level class observations displayed in the CrowdWater game are obtained from the CrowdWater app (see Fig 1 and 2.1 The CrowdWater app). The CrowdWater game uses all water level class observations with a picture and displays each picture together with the reference picture for that site (Fig 2). By February 28, 2019, there were 2326 picture pairs in the game but this number is increasing continuously as the app users continue to create new spots with reference pictures and provide observations and pictures for existing spots. These pictures are automatically transferred to the game. The game players compare the picture pairs and estimate the water level class for the picture of the new observation (i.e. the one that does not have the virtual staff gauge). This way many players can assess the same situation without being outside at the same stream at the same time and thereby peer-review the water level class data that are submitted via the app. As the players only get to see a photograph, rather than seeing the actual stream, we need to test the value of the game for data quality control and how many votes need to be collected per observation. Obviously, if a water level observation is uploaded without a new picture (< 3% of all observations), data quality control via the game is not possible.

#### 2.2.2 Observation reports

While playing the game, players can use a report button (Fig 2) when they believe that the water level cannot be determined for the new picture or when there are other issues with the pictures. Players can choose one of six reasons to report an observation. In the case of *“other reason”* the player can write what that reason is.

- The photo is ok, but I don’t know the category.
- The staff gauge is not placed correctly.
- The staff gauge is missing.
- The approved value is clearly incorrect.
- The location has changed and the reference image is unrecognizable.
- Other reason: …

#### 2.2.3 Gamification aspects of the CrowdWater game

In its current implementation, the CrowdWater game has monthly championships that consist of 28 daily rounds. Twelve picture pairs are shown to the players per round. There are two different types of picture pairs: classified and unclassified observations. Classified observations have already received 15 or more votes by at least 15 different players. On February 28, 2019, there were 846 classified observations (i.e., classified pictures) in the game. Within the game, the median of the votes for the water level class for a classified observation is calculated and used as the approved value to which the vote of the current player is compared. Currently, the classified observations remain in the game until they have received 100 votes. When there are fewer than 15 votes for a picture, it is defined as *“unclassified”* and, thus, does not have an approved value assigned to it yet. On February 28, 2019, there were 1480 unclassified observations in the game. The players do not know which type of observation they are looking at until they have voted for a water level class. This is a similar approach as for the Cropland Capture game [17]. For already classified observations, players receive six points if they choose the same class as the median of the votes from the other players (considered to be the approved class), four points if they choose a neighbouring class and zero points if they are more than one class off from the median. For (so far) unclassified observations, players always receive three points (regardless of their vote). When reporting a problem for a picture pair (see section 2.2.2 Observation reports) players always receive three points so that there is no incentive to try to classify a picture that should be reported. The distribution of points also ensures that it is not possible to win a round merely by reporting every observation.

After completing a full round of pictures in the game, the players see the score and all twelve picture pairs together with his/her vote and the approved water level class for each picture pair. This provides feedback to the player on what the correct water level class was. At this stage it is possible to report a picture again (but this time this does not lead to any points).

The names of the top ten players for the daily rounds are shown on the leader board (Fig 2). Every month a new championship starts and small prizes are given to the overall winners of the monthly championship (i.e. the three players with the most points for that month), as well as three randomly selected players who won at least one of the daily-rounds during the championship.

#### 2.2.4 CrowdWater game participants

The game was tested internally (i.e., within our research group) between May and July 2018 and has been published and promoted online since August 2018. The game was advertised through several communication channels: the CrowdWater homepage, facebook, twitter, LinkedIn, ResearchGate, CrowdWater app push-messages, CrowdWater newsletter, SciStarter, Schweiz Forscht, citizensciencegames.com, as well as by directly contacting colleagues, friends and family.

The frequency distribution of the contributions per player in the CrowdWater game is similar to many other citizen science projects [5,48–51]: there are a few dedicated participants who play very frequently and contribute the majority of the votes, whereas many participants have only tried the game once or play infrequently (see 3.1 Participants).

### 2.3 Analysis of the CrowdWater game data

#### 2.3.1 Correction of app data through the game

The aim of the game is to check the water level class data submitted through the CrowdWater app and if necessary correct the water level class observations. For each observation that has been classified by 15 or more players in the game, we computed the mean water level class from all the game votes. To exclude the influence of outliers, only the values within the 10^th^ and 90^th^ percentiles were used to compute the mean. This mean value differs from the current implementation of the CrowdWater game, where the approved water level class is determined based on the median value. The discrepancy between the analyses in this paper and the implementation in this game is due to the game being implemented earlier; we expect that the game will be adapted in the near future so that it also uses the mean water level class as the approved value as this more accurately reflects the correct water level near class boundaries (see results section 3.2 Vote distribution per observation and data correction). The difference between the mean water level class from the game and the water level class submitted via the app can be divided into three categories: no discrepancy, a water level class correction, and a higher water level class resolution.

- *No discrepancy*: If the mean game vote is within < 0.25 classes from the original value submitted via the app, no correction of the original app value is necessary.
- *Water level class correction*: If the mean value of the game votes is more than 0.75 water level classes away from the value submitted via the app, either the original app value or the mean game vote needs to be corrected.
- *Higher water level class resolution*: The water level of a stream can be at the border of two water level classes. In the app the citizen scientist has to decide on one of these neighbouring classes. In the game, the player also has to decide on one of the classes, but based on the distribution of the votes from many players it sometimes becomes apparent, that the actual water level class is in between the two classes. Therefore, if the mean game vote is between 0.25 and 0.75 classes away from the value submitted via the app, it could be considered as a half-class, i.e., a value with a higher resolution of the water level class scale than is possible in the app.

If the water level class submitted via the app and the mean game vote do not agree (i.e. they differ by more than 0.75 water level classes), expert judgement is needed to determine which of these values is most accurate. Experts can decide based on the pictures of the stream level that are also shown in the game whether the app value or the mean game vote is more likely to be correct. Two of the authors (Strobl and Etter) checked and classified the observations individually and discussed the pictures in case their expert judgement differed. The expert judgement was only done to evaluate the performance of the CrowdWater game, to better understand the accuracy of the output of the game for future applications. This will not be done continuously for the CrowdWater game as the number of pictures that need to be assessed would quickly become unmanageable. The categories used for expert judgment were as followed:

- The original app value was correct.
- The mean game vote was correct.
- Neither was correct, but the original app value was closer to the correct value.
- Neither was correct, but the mean game vote was closer to the correct value.
- The correct value was precisely in the middle between the original app value and the mean game vote.
- The observation should have been reported, rather than voted on by players, e.g. because there was no possibility to determine the exact value based on the picture.

#### 2.3.2 Vote distribution per observation

For each observation that had at least 15 votes, we determined the distribution of the differences between each vote and the mean game vote (i.e. the error distribution). The distribution of the errors is an important indicator of the certainty of a mean value and showed how sure *“the crowd”* was of their collective vote. We wanted to know if the error distribution of the game votes was similar to the error distribution of the water level classes for people who see the actual stream (rather than only a picture). We therefore compared the error distribution for all observations in the game with the error distribution from a previous field study, in which 517 passers-by at ten different locations were asked to estimate the water level class of the stream by comparing the current situation with a printed copy of the reference picture with the staff gauge [45]. The error distributions for the game players and the passers-by were not normally distributed, therefore we used the Mann-Whitney test to compare the medians of the two datasets and the Pearson Chi-Squared test to compare the frequency distributions.

#### 2.3.3 Impact of the number of votes on the calculation of the mean water level class

We wanted to determine the number of votes per observation that are needed to obtain a correct mean game vote to be able to design the CrowdWater game in such a way that it accurately classifies the observations in the most efficient way possible (i.e., to remove observations from the game when they have been classified by a sufficient number of game players, so that the effort can be directed towards classifying new observations). Currently 15 votes are needed to classify a picture but this number was merely an initial guess and could be changed based on the results of this analysis. We used bootstrapping to evaluate how the number of votes per observation affects the uncertainty of the mean game vote and thus the resulting water level classification. More precisely, we compared how the mean value of the votes for a randomly chosen subset of votes (ranging from 1 to 30 votes) for each classified picture differed from the overall mean that takes all votes into account. We then determined the number of classified pictures for which this difference was less than 0.05 and less than 0.2. This was repeated 10 000 times. The analysis was only done for classified pictures with at least 30 votes (246 observations or 11% of all observations). For this analysis we used the actual mean vote, rather than the mean within the 10^th^ to 90^th^ percentile of the votes, as the exclusion of outliers was not practical for the smaller subsets of votes.

#### 2.3.4 Accuracy per player

We also tested whether there are differences in the abilities of the players to classify an observation correctly and if this is connected to how regularly they play the game, i.e., whether regular players are better at assigning the right water level class to an observation than novice players. Therefore, we calculated the mean accuracy per player, which is the mean of the absolute difference between the vote of the player and the mean game vote (within the 10^th^ to 90^th^ percentile of all votes) for all of their votes and subtracted this value from 10 (the maximum possible divergence). Thus an accuracy score of ten indicates a perfect score (i.e., the votes of the player were always the same as the average vote from all players), whereas a low value indicates that the votes of the player were often different from the average vote. To check whether or not the mean accuracy per player was significantly different for the regular and novice players, we used the Mann-Whitney test (p < 0.05) because the Shapiro-Wilk normality test indicated that the mean accuracy values were not normally distributed. Regular players were defined as players who played more than two full rounds of the game (38% of all players, representing 96% of all votes), whereas novice players played fewer rounds.

### 2.4 Survey

To address questions related to the motivation of the participants, we sent out a short survey to everybody who had played the CrowdWater game at least once before 11.02.2019 (145 players), using the email addresses that were used to register for the game. The questions in the survey took 5–10 minutes to complete and covered several topics, such as what motivated the respondents to play the game, which game aspects they liked, and which ones were frustrating. In the survey we also asked if the respondents had used the CrowdWater app and how their experience with the app compared to their experience with the game. The full survey can be found in the supplementary material 1 (S1 File).

## 3. Results

### 3.1 Participants

By 28.02.2019, 153 players had registered for the CrowdWater game and contributed at least one vote; in total, 33 176 picture pairs have been compared. However, only 58 players had played more than two rounds, indicating that many participants only tried the game once or twice. The average number of observations classified per participant was 148, but the median was only 12. The mean number of classifications for the five most dedicated contributors was 1829. These results indicate a very skewed distribution of the number of classifications among the participants. Few of the participants who participated in the survey had watched the tutorial movie (36%) but more participants read the manual (61%) before playing the game for the first time.

### 3.2 Vote distribution per observation and data correction

**Fig 3:**
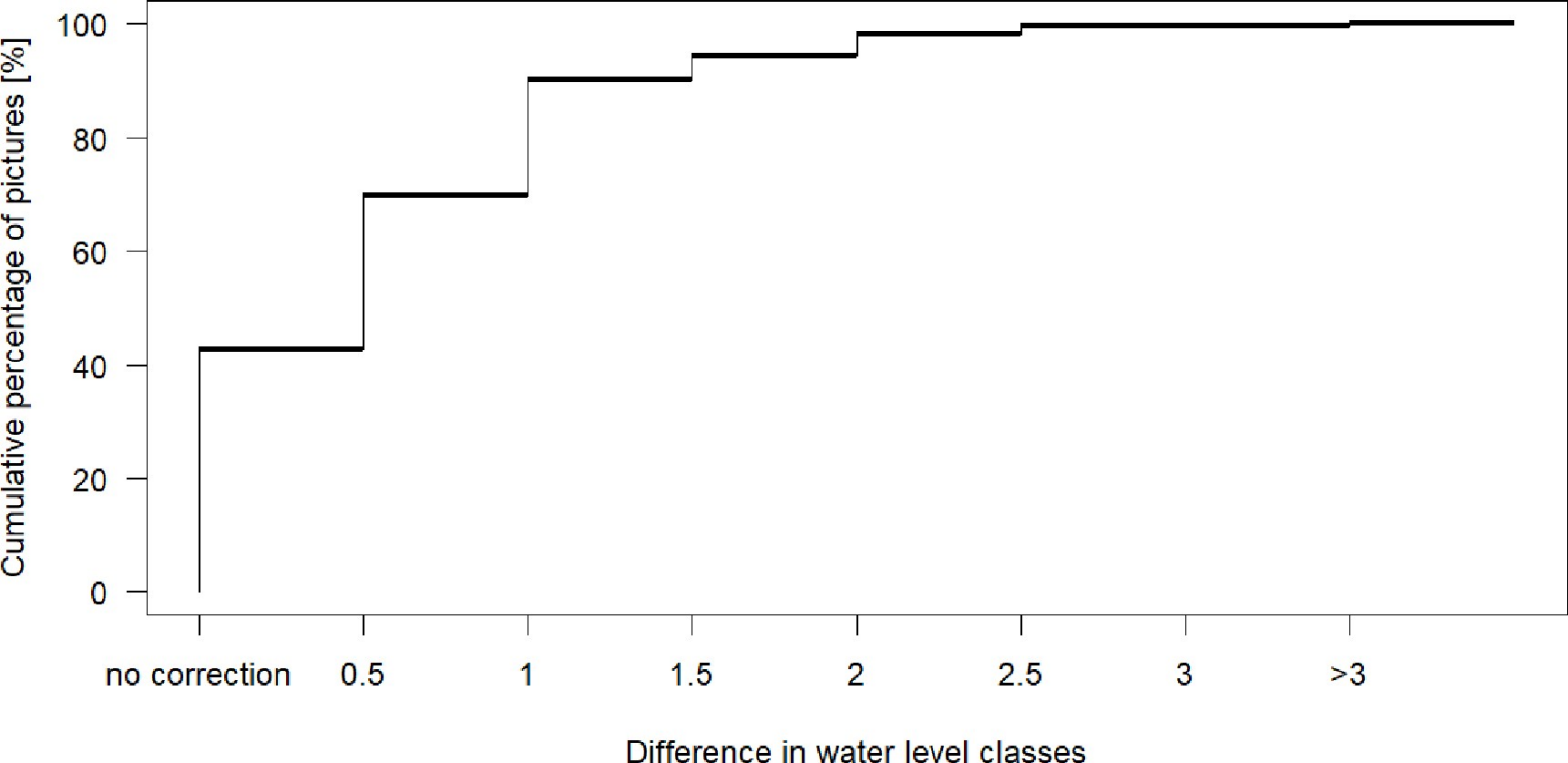
Cumulative frequency distribution of all corrections. Corrections of the original app value based on the mean game votes (between the 10^th^ and 90^th^ percentile). 100% = 841 classified observations.

The agreement of votes for classified pictures varied significantly from observation to observation and sometimes even for observations taken at different times for the same site. However, the app values and the mean game vote rarely differed by more than one water level class. For 43% of all classified observations there was no difference between the original water level class submitted via the app and the mean game vote, meaning that the app user and the mean vote of the game players agreed. For 27% of all observations, the mean value from the game differed by half a water level class, which should not be considered an error, but rather an increase in the resolution of the data (i.e. indicating a water level between two class boundaries). For 30% of all classified observations the mean game vote and the app entry differed by at least one class. For 20% of all classified observations the disagreement was exactly one class; for only 10% of the classified observations, the mean game vote and the original app value differed by more than one class (Fig 3).

**Fig 4:**
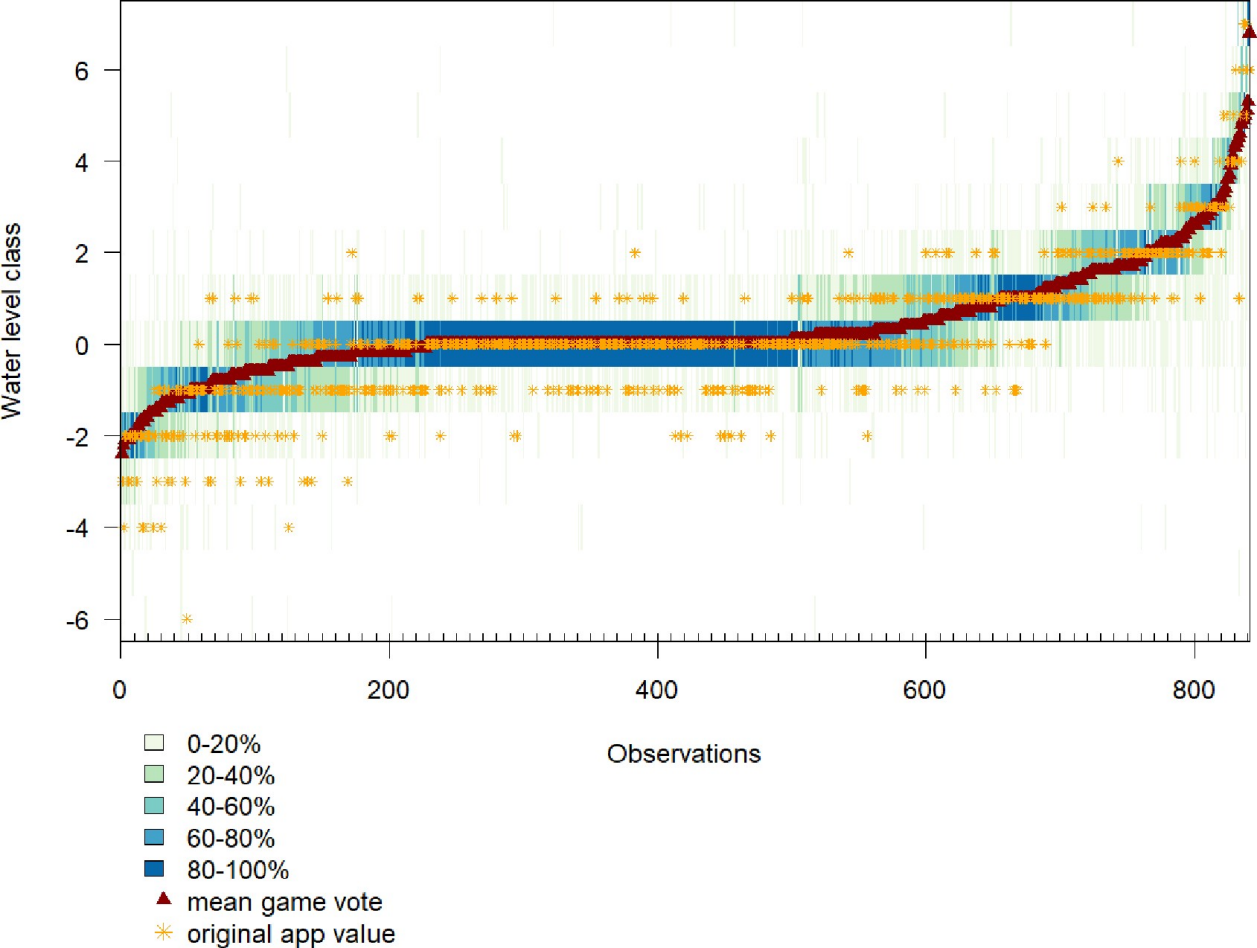
Agreement among players (in %) per classified observation. Each column represents one observation, sorted according to the mean game vote (red triangle). Darker colours represent a higher agreement and lighter colours a lower agreement among the players. The original value of the water level class submitted via the app is indicated by the orange star.

The agreement among the game players was particularly high for observations for which the water level was relatively similar to that in the reference picture (i.e., the mean vote had a water level class of zero; Fig 4).

**Fig 5:**
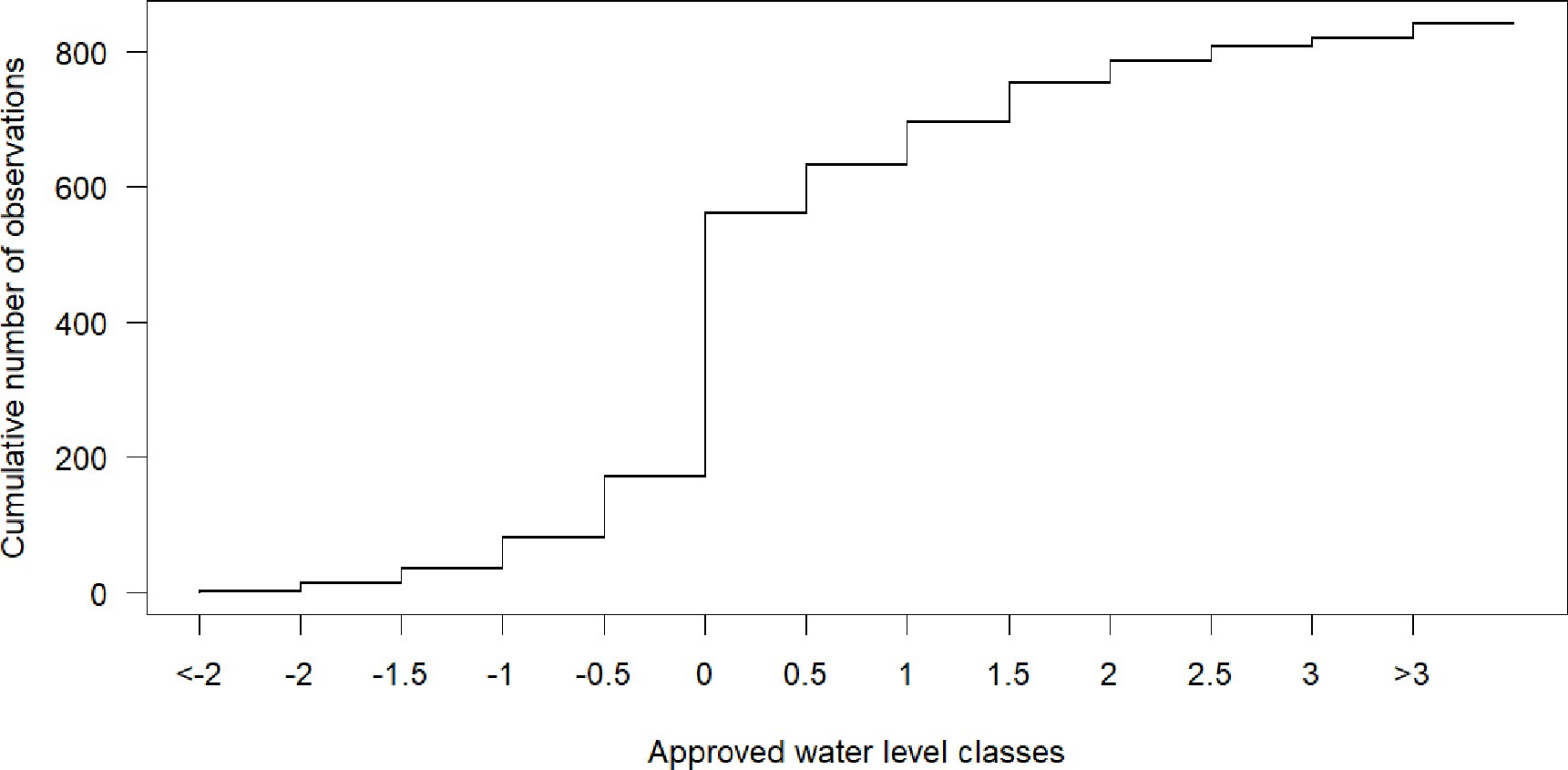
Cumulative number of classified observations per water level class based on the mean game value.

Based on the mean game value, 390 of all 846 classified observations (46%) fell into water level class 0 (i.e., similar to the water level class as in the reference picture), whereas all other classes had < 10% of the observations respectively. For 263 (31%) classified observations the mean vote indicated a half-class (Fig 5).

For 77% of the observations with a water level class of +1, the mean vote was class +1 (n = 64), whereas 68% of the observations with a water level class of -1 had a mean vote class -1 (n = 45).

**Fig 6:**
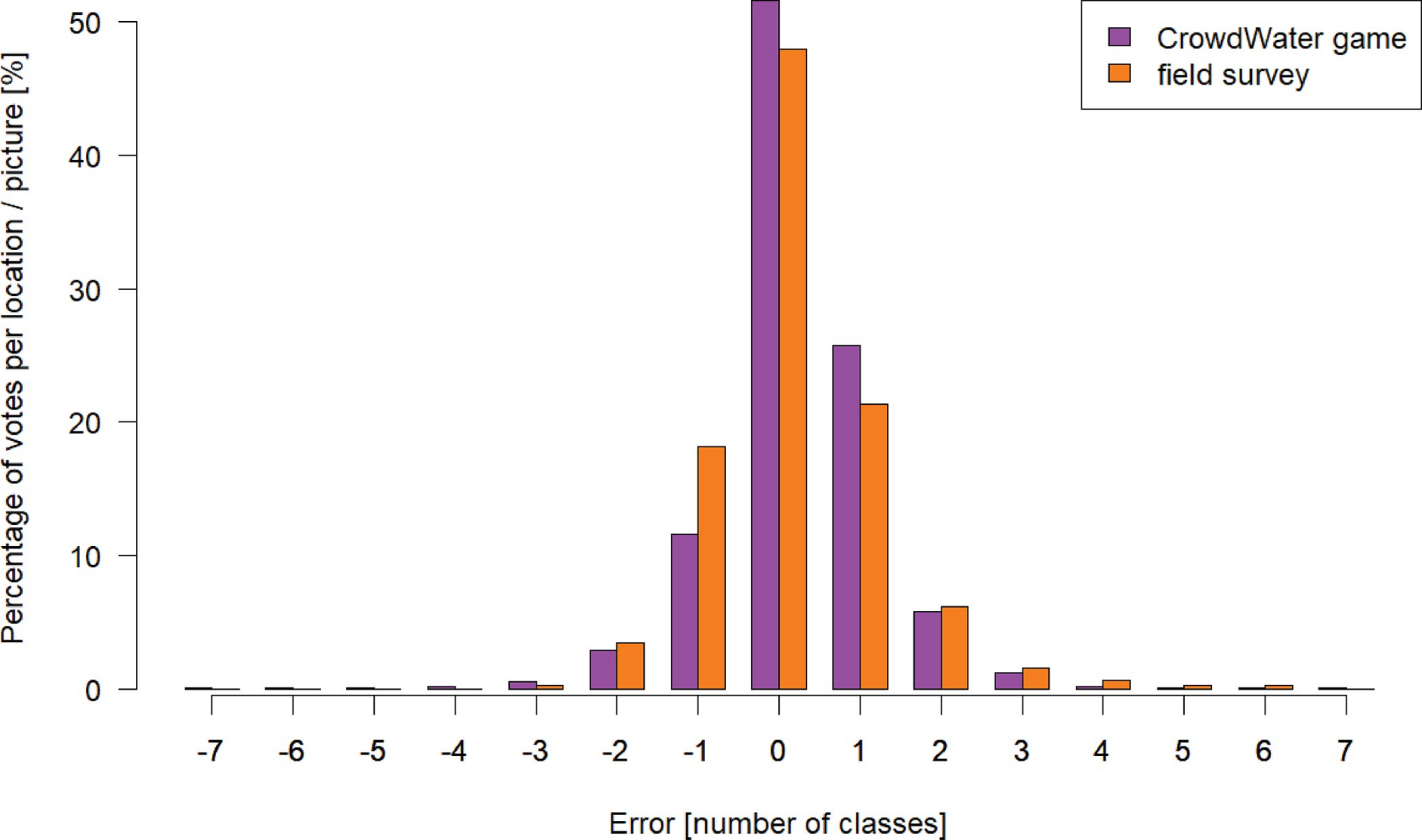
Error distribution of the water level classes for the CrowdWater game and a field survey. Comparison of the error distribution of the votes in the CrowdWater game (difference between the vote of a player and the mean game vote for that observation; n = 841) and for previously held field surveys (n = 517) [data from 49].

The frequency distribution of the differences in the votes per observation from the mean game vote for that observation was similar to the distribution of the errors in the water level class assignment for 517 passers-by who estimated the water level for ten streams with the same virtual staff gauge approach [45]. For both the game and the real life situation more than 48% of the participants chose the right class, and less than 3% were more than two classes off. The median difference in the water level class values (0 for the game and the passers-by) and the frequency distribution were not significantly different either (p < 0.05; Fig 6). The accuracy was comparable, as the two distributions were not significantly different from each other (based on a Pearson Chi-Squared test, p < 0.05).

All 252 observations for which the mean game vote and the original app value differed by one or more class, were classified through expert judgement. For 60% of these cases the mean game vote was considered to be correct and for 14% of cases neither the mean game vote nor the app value was correct, but the mean game vote was closer to the correct value. For 8% of these cases the app value was correct and in 1% of cases neither were correct, but the app value was closer to the correct value. For 8% of cases the correct value was precisely between the app value and the mean game vote. For 9% of the cases the observations should have been reported, rather than getting a water level class vote.

### 3.3 Accuracy per player

**Fig 7:**
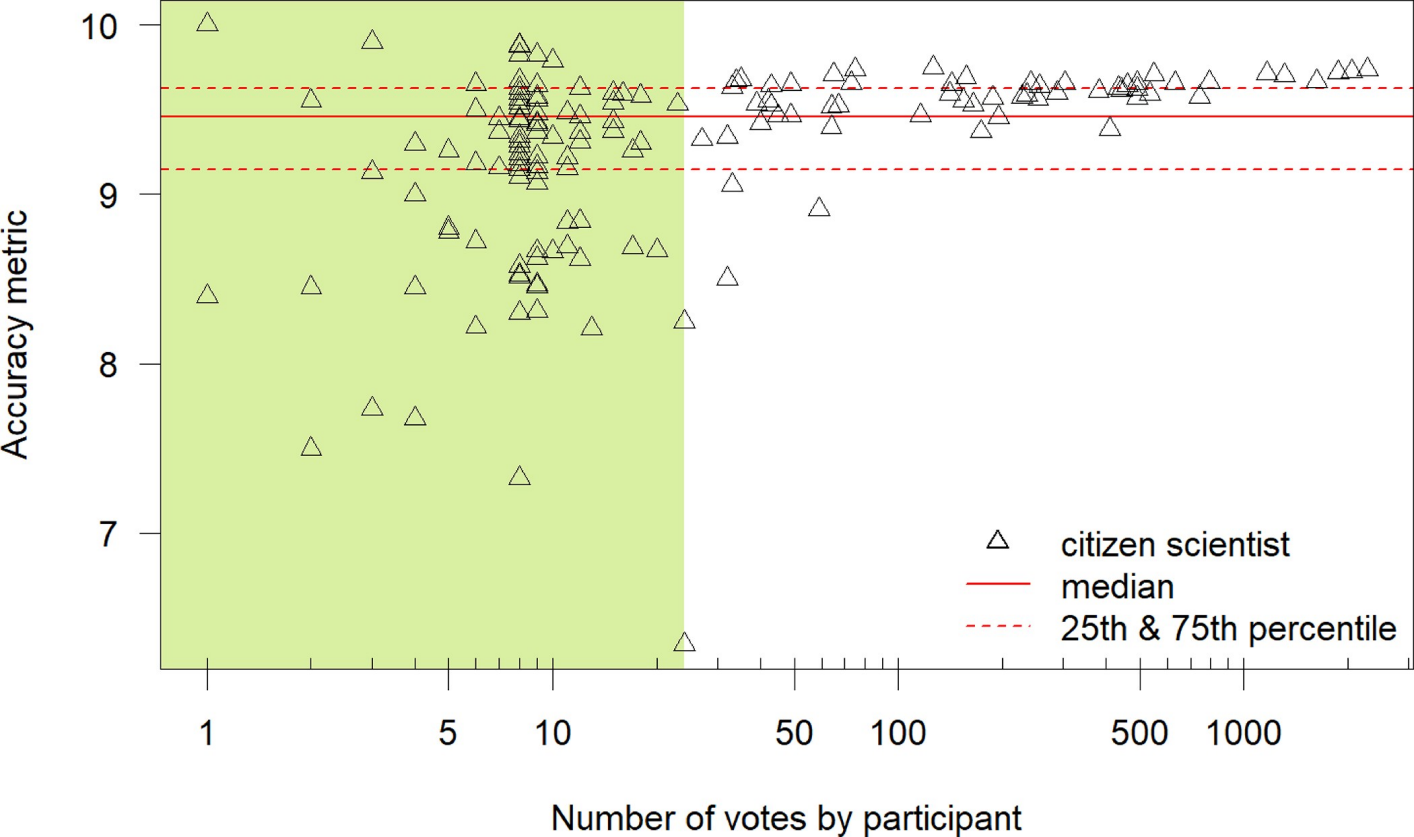
Mean accuracy per player. Mean accuracy per player as a function of the number of observations that that player classified (each triangle represents one player). The lines indicate the median accuracy for all players (solid line) and the 25^th^ and 75^th^ percentile (dashed lines). The green shading indicates the novice players who played a maximum of two rounds (24 classifications). Note the log scale on the x-axis.

The median of the mean accuracy per player for the 58 regular players (i.e. > 24 classifications per player) (9.60) was significantly better than the median of the mean accuracy per player for the 94 novice players (≤ 24 classifications per player) (9.26). The range in the mean accuracy per player was smaller for the regular players as well (1.24 classes for the regular players vs. 3.63 classes for the novice players). Players seem to get even better with more rounds, as the median for players with more than four rounds (9.62) was also significantly better than for players between two and four rounds (9.53). All very frequent players (those who classified > 500 observations; n = 11) had a mean accuracy that was higher (i.e., better) than the median of the mean accuracy for all players (Fig 7), however statistical differences could not be calculated due to a small sample size of very frequent players.

### 3.4 Observation reports

Almost half of the players used the report function at least once. Of all players who reported at least one observation, 77% were regular players, or, expressed differently 35% of the regular players and 87% of the novice players never reported an observation.

**Fig 8:**
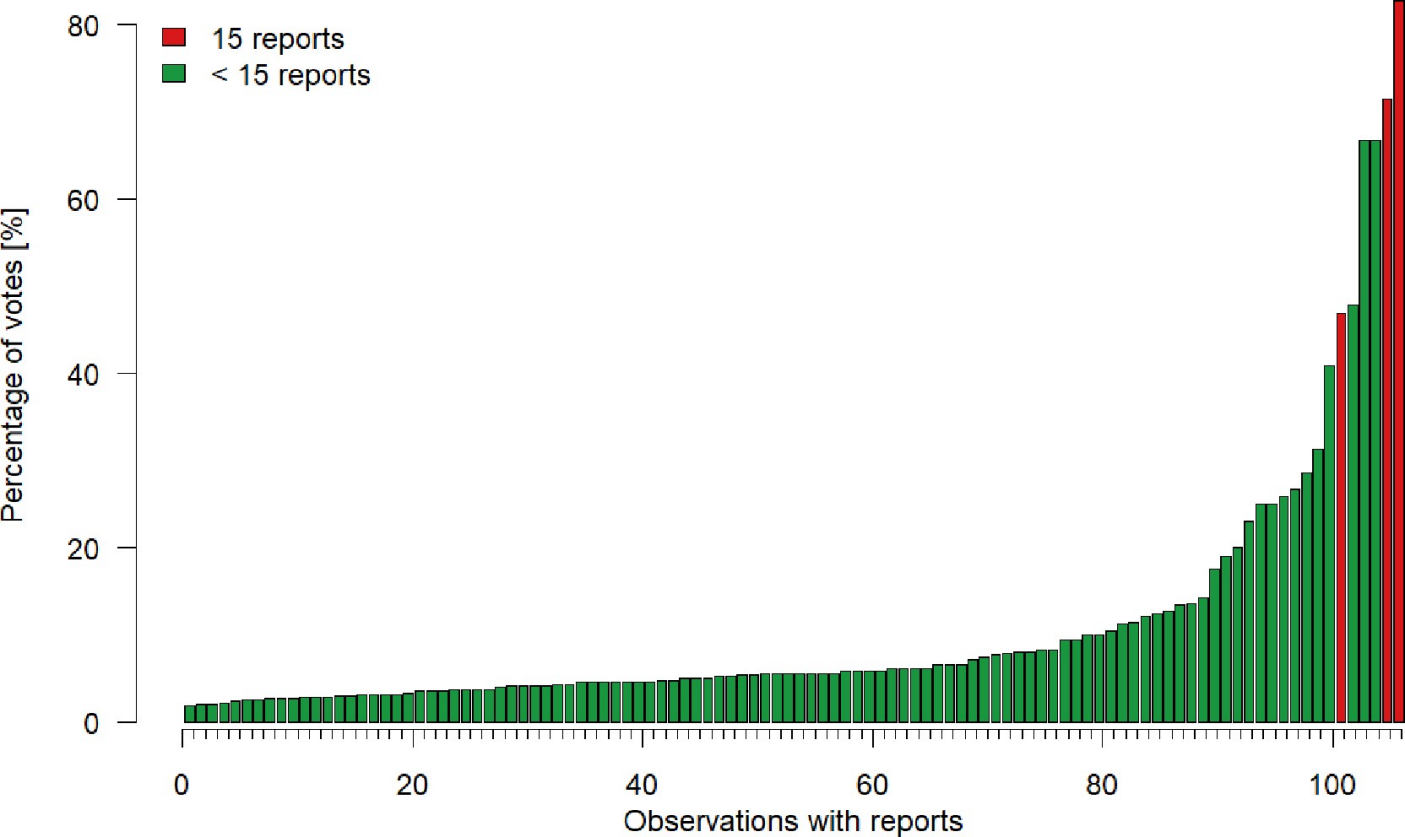
Percentage of reports over all votes. Reports for each of the 841 classified observations (i.e. at least 15 votes in total) that received at least one report. The red colour indicates that the observation received 15 reports, after which it is excluded from the game.

The report function was used at least once for 8% of all observations (classified and unclassified) that were in the game (n = 193). After an observation has received 15 reports, it is removed from the game. So far this occurred for only three observations (Fig 8).

The most common reason for reporting an observation was *“other reason”* (47% of all reports). Within this category, 48% of all reports stated that the picture was too dark. The second most common report was *“the location has changed and the reference image is unrecognizable”* (32%), followed by *“The staff gauge is not placed correctly”* (10%) and *“The staff gauge is missing”* (10%). The other report categories were rarely used (< 3% each) (Table 1).

**Table 1:** Reasons given for a report as a percentage of the overall number of reports.

Report reason	Percentage of reports
The photo is ok, but I don’t know the category.	1%
The staff gauge is not placed correctly.	9%
The staff gauge is missing.	9%
The approved value is clearly incorrect.	2%
The location has changed and the reference image is unrecognizable.	32%
Other reason: too dark to classify	23%
Other reason: all other reasons	24%

### 3.5 Impact of the number of votes on the mean game vote

**Fig 9:**
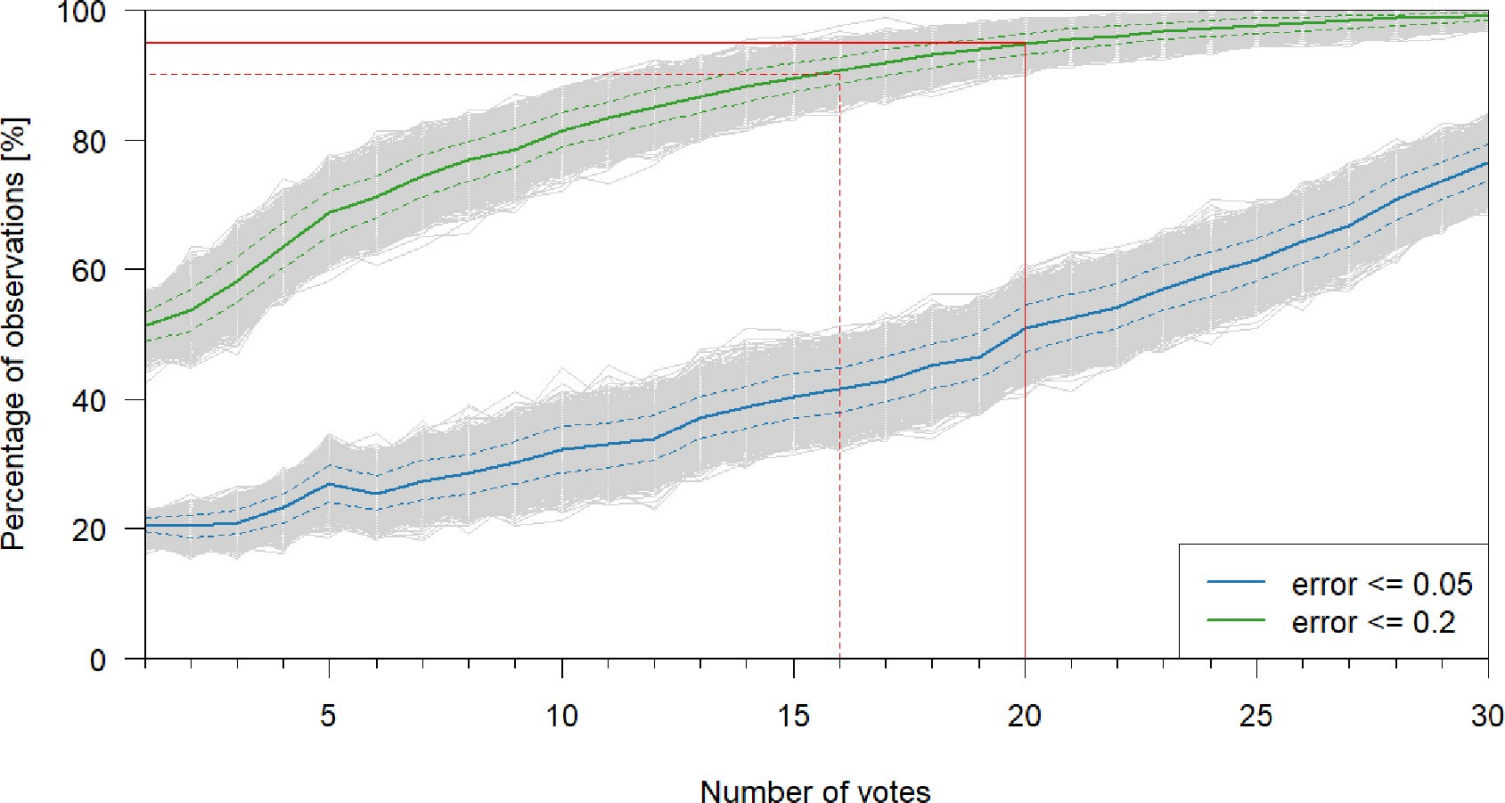
Impact of number of votes. Percentage of observations with an error ≤ 0.05 (median in blue) or an error ≤ 0.2 (median in green) as a function of the number of votes per observation. The dashed blue and green lines indicate the 10^th^ and 90^th^ percentile, while the grey lines show the results for all 10 000 iterations. The solid red lines indicate that for 90% of observations the error is ≤ 0.2 after 16 votes. The dashed red lines indicate that for 95% of observations the error is ≤ 0.2 after 20 votes.

The impact of the number of votes on the mean of the game votes for that observation shows at what point the mean game vote becomes stable, i.e., the mean value does not change with additional votes. The results of the bootstrapping analyses indicate that for 89% of observations the error was ≤ 0.2 after 15 votes, for 90% of observations the error was ≤ 0.2 after 16 votes and for 95% of the observations after 20 votes (median values for all 10 000 iterations) (Fig 9). An error ≤ 0.2 would still be rounded to the approved water level class. More votes steadily increased the percentage of observations above these thresholds.

### 3.6 Survey

A quarter of all players [36] who were sent the link to the survey filled in the survey. Half of the respondents of the survey played the CrowdWater game at least once a week, a quarter of the respondents played the game one to three times per month, and another quarter of the respondents had played only once.

**Fig 10:**
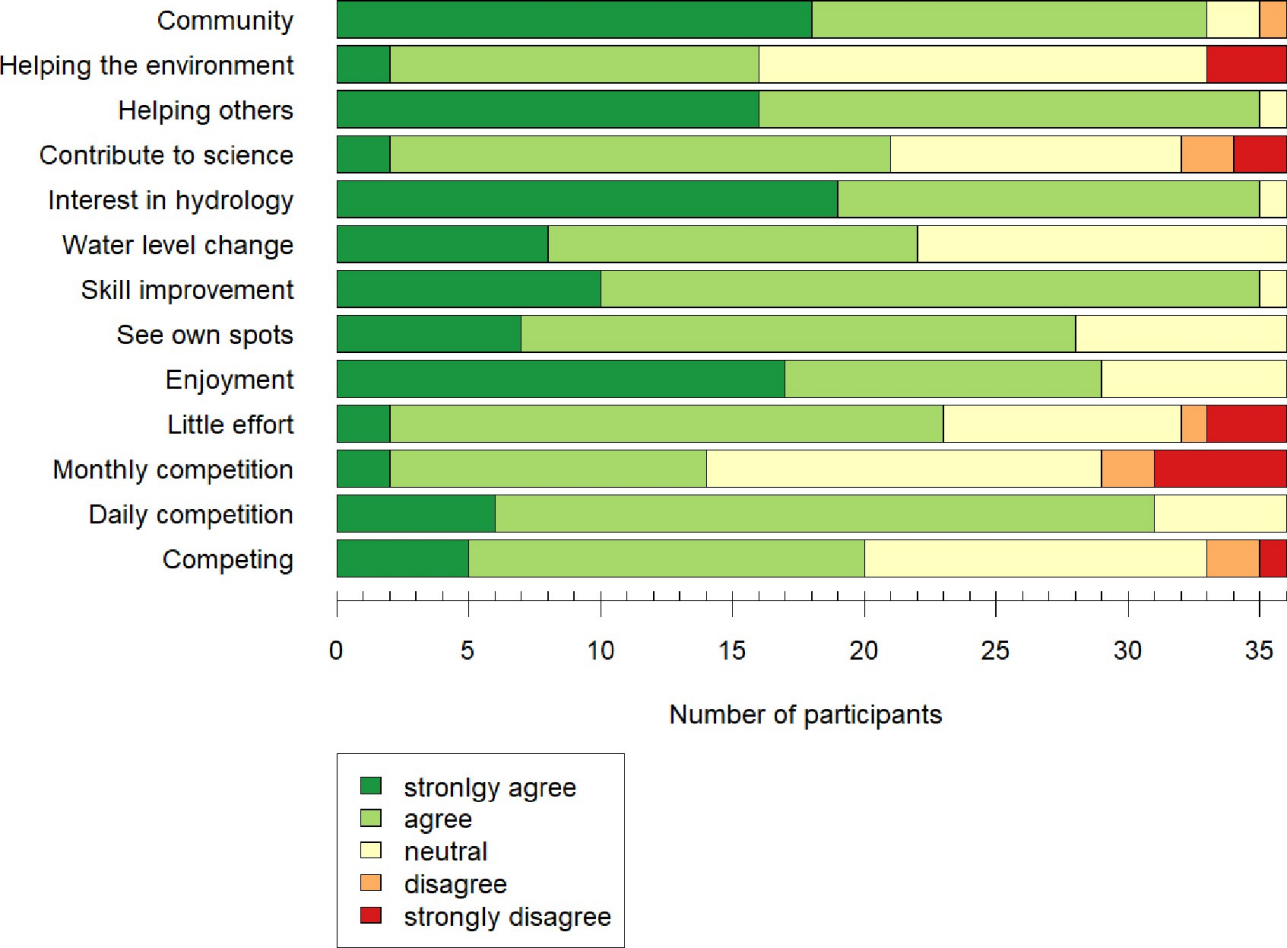
Motivation of the participants to play the CrowdWater game. The survey questions can be found in supplementary material 1 (S1 File).

The main motivations for playing the game were enjoyment in playing the game, a general interest in hydrology, and being part of the CrowdWater community. Contributing to science, helping the environment, and the monthly competitions were less frequently mentioned as motivating factors (Fig 10). The majority of respondents stated, that they enjoy *“classifying difficult pictures*, *even though I might not get full points”* compared to a minority who said that they enjoyed *“classifying easy pictures*, *because I will likely get full points”*. Almost two thirds of respondents said that they enjoyed *“competing against each other”*; one person found that *“the points and competition are unnecessary and distract from the scientific goal”*. Overall the three aspects found to be most frustrating and marked by about half of all respondents were *“difficulty finding adequate references in the pictures”*, *“pictures that are not taken from the same angle”* and *“not getting full points*, *even though I am sure of my vote”*.

The majority (67%) of the players who responded to the survey also use the CrowdWater app. Almost half of them enjoyed both activities equally, a third enjoyed using the app more, while a quarter enjoyed the game more. The large majority (79%) of respondents who also use the app indicated that the game helped them *“be more aware of how to place a staff gauge in the app”*. Half of the respondents stated that the game helped them *“to estimate water level classes in the app”*.

## 4. Discussion

### 4.1 Can peer-review improve the quality of crowdsourced water level class data?

Many publications have reported on the accuracy of citizen science games, with game aims, topics and styles covering an extensive range. Therefore the results and the accuracy metrics used in these previous publications vary considerably. Generally the conclusions have been very positive and many of these citizen science games are still online and collect valuable scientific data [24,35,37,49,52,53]. The results of the CrowdWater game are also positive and suggest that showing the same picture pair to multiple players and taking their collective vote as the approved value can help improve the quality of the water level class data that are collected by the CrowdWater app. A big benefit of peer-review compared to a data filter, is that incorrect data are not only filtered out but can be updated and therefore can still be used as a valid data point in later analyses. Furthermore, it does not require pre-defined criteria of what data are likely to be incorrect.

For 70% of all water level class observations, there was either no difference or a difference of only half a class between the mean game vote and the original app value. For 74% of the observations for which there was a difference between the mean game vote and the original app value, the game provided a more accurate estimate of the water level class than the app. This suggests that the game is a valuable tool to check the quality of the water level data provided through the app. We would therefore strongly recommend collecting pictures together with any citizen science data (if feasible), even in projects where this does not seem essential at first.

The frequency distribution of the vote errors in the field survey was similar to that of the CrowdWater game, even though the reference pictures in the field survey were all initiated by us (which should have guaranteed a reasonable quality) and the participants were able to look at the stream directly. In the game, the reference pictures are created by citizen scientists using the app (where sub-optimal reference pictures do occur) and the player can only take a look at a picture of the stream. The sense of depth may be different for the picture than when one sees the actual stream. In spite of these differences, and even though not all pictures are optimally sharp or free from distortions, citizen scientists were just as good at determining the water level class in the game as the passers-by that assessed the water level class outside. This furthermore suggests that it is beneficial to obtain pictures with the submission of the data via the app and to use these pictures in a gamified approach to improve data quality.

However, in some cases (9% of all observations for which the mean game vote differed from the water level class submitted via the app), the water level class obtained from the mean game vote was less accurate than the original app value (as determined by expert checks). When the number of observations increases, it will be impossible to determine whether the mean game vote or the original app value is right. It would be useful to automatically identify these cases, to avoid changing a correct app value. To identify these observations, indicators could be used, such as kurtosis, as a proxy for the vote distribution, bimodality to indicate diverging interpretations of the same picture, location to indicate a poorly placed staff gauge in the reference picture or the overall contribution of the app user or mean accuracy of the contributing game players [54]. Currently not enough data are available to assess, whether these indicators can be reliably used, however, in the future this topic might be worth revisiting. Another option to assess the correctness of the original water level class submitted via the app would be to go back to the observer who submitted the original via the app and to ask how certain (s)he is about the initial estimate. In some cases, such as accidental misclassification, the discrepancy might be easily resolved. Additionally this would provide feedback to app users, which might provide training and increase the accuracy of their data. By receiving feedback app users find out that their data are checked. This could increase their confidence in being able to contribute high-quality data, which was discovered to be useful in a study where citizen scientists from seven different online citizen science projects were interviewed [5]. If the app user insists that his/her water level class estimate is correct, the observation could be reviewed by an expert, as this would likely occur for fewer cases than all corrections. Currently, without any further means of validating the game results, it still makes sense to use the mean game vote as the approved value, as in 74% of cases the game provided a more accurate water level class than the original submission via the app.

### 4.2 Can the vote distribution per observation provide additional information?

The primary reason for using several votes per observation was to minimise errors in the water level class assignment because the errors of individual game players likely average out (i.e. wisdom of the crowd [55]). The vote distribution per observation shows if there is a high agreement between players, which suggests a higher certainty for the resulting mean game vote, and thus higher certainty for the water level class. The results showed that the highest agreement between players is at class zero, which per definition represents a situation that closely resembles the reference picture. Some of the uncertainty for very high or low water level observations can therefore partly be derived from the interpretations that game players make to assess a very different looking picture. The interpretation is particularly tricky, if the correct water level class is below the water surface in the reference picture, as the relevant reference features might not be visible. This is supported by the results indicating a higher percentage of votes for the approved class for observations with class plus one compared to class minus one (77% vs. 68%).

The vote distribution per observation can also indicate if the water level is at the boundary between two classes. This results in higher resolution water level class data than is possible in the app. The app user has to decide on one of the two classes, even if the user believes that the water level is exactly at the border of two classes. While the individual game users also had to decide on either the upper or the lower class, their vote distribution could show that the water level was likely in the middle of two classes. This higher resolution water level class data may be useful for further analyses of these data, e.g. to calibrate a hydrological model.

For a few observations, we detected a bimodal distribution in the game votes (not between two neighbouring classes), which could indicate that a picture was unclear and allowed for multiple interpretations. These observations all came from the same location, which had large changes in the amount of sediment in the stream bed after the reference picture was taken (both deposition and scour). Based on this small sample, it is not possible to conclude that a bimodal distribution only occurs for such scenarios and can be used to filter out such situations. However, similar results regarding the agreement among players were found in a study based on cropland identification, where *“crowdsourcing appeared particularly cost-effective in areas that were easy to interpret and allowed difficult or problematic sampling units to be identified*, *i*.*e*., *as evidenced by a lack of consensus between volunteers*.*”* [54].

### 4.3 Are votes from regular players more accurate?

The votes from regular players, were statistically significantly more accurate than the votes from novice players. There are two possible reasons for this higher accuracy. On the one hand players might get better after playing several rounds and therefore their mean accuracy improves. On the other hand, novice players might notice that they consistently get few or no points and therefore drop out of the game. The survey showed that only a minority of the players really cared about achieving a high score, but nonetheless some stated that they found it frustrating to vote for difficult pictures, as they might not get full points. Perhaps the players who got few points and dropped out of the game after a few rounds were the same ones that were eager to get many points and win, however, we do not have data to check this assumption.

A similar difference in accuracy for regular and novice players was not observed for the Forgotten Island and Happy Match games, and only a minimal difference was found for the game Happy Moths [49]. However, for the Cropland Capture game, it was shown that the score of players could indeed improve over time [56]. This suggests that the difference in the accuracy of the votes between regular players and novice players and the potential for improvement of the accuracy when playing the game depends on the game.

There is also the possibility of bias in the game. Observations had to be classified by 15 different players before they were considered classified. Due to game logistics, further classifications were not necessarily done by different players. Therefore, it is possible for the same player to classify an observation more than once, thereby influencing the mean game vote which results in a higher accuracy. However, due to a large number of players and many observations available in the game, we assume that this effect is negligible.

### 4.4 Can players correctly identify unsuitable observations through the report function?

The report function is a valuable tool to filter out unsuitable observations. When an observation is reported because *“The staff gauge is not placed correctly*.*”*, *“The staff gauge is missing*.*”* and *“The location has changed and the reference image is unrecognizable*.*”*, the observation should not only be removed from the game, but also from the app, as e.g. a reference picture without a staff gauge cannot be used to obtain a water level class time series. For the categories *“The photo is ok*, *but I don’t know the category*.*”*, *“The correct value is clearly incorrect*.*”* and *“Other reason”* the observation and picture might not be suitable for the game, but might still be a valuable data point. The reported reason *“the picture is too dark”* is an example of a picture being unsuitable for the game, but the app player might still have seen the location adequately to estimate the water level class correctly. On the other hand, the report reason *“The location has changed and the reference image is unrecognizable*.*”* indicates a problem with the reference picture or it might be challenging to find a suitable reference. Here the number of votes could be used as an indication: if the majority of players reported the observation it is likely that there was an issue with the reference picture, but if only a few players reported the observation it might simply be difficult to find a suitable reference. The observation in the app should be removed, if the streambed has indeed changed significantly between the time that the reference picture was taken and the new observation.

The fact that roughly half of all players never used the report function, could indicate that these players are unaware of the report button or are unsure when to use it. This is also supported by the fact that only very few novice players (13%) used the report function at least once (compared to 65% of regular players). The infrequent use of the report function was also reported for the Cropland Capture game, which has a button so that players can choose *“maybe”* instead of stating whether a picture displays cropland or not. This button was only used infrequently (rarely over 50% of the votes per picture), even for pictures that were difficult to classify [34].

If a large number of players cannot find the report button in the CrowdWater game, it may take longer for certain spots to be removed from the game. There were several ambiguous cases, when it might still be possible to guess the water level class on a relatively dark picture, but the estimate was likely uncertain. In these cases some users opted for the report button and others decided to vote for the most likely water level class. In addition, there was a chance that players just guessed that the water level class is zero (which is the most frequently occurring water level class) in order to get full points.

### 4.5 How many votes are necessary to achieve a stable mean?

Our current number of 15 votes per observation to consider an observation classified and keeping it in the game for 100 votes seems to work well because for 90% of the observations the error was ≤ 0.2 after 16 votes. Allocating game points with a certainty of just under 90% seems sufficiently accurate, especially as more votes are collected overall. We will therefore leave the classification threshold at 15 votes for the point allocation, but will reduce the total number of votes per observation to 50, in order to more quickly complete the classification of observations within the game to more quickly classify observations.

Other projects have investigated the ideal number of votes in a similar way. The cut-off number for votes varies depending on the citizen science project but is comparable to the cut-off value found for the CrowdWater game. The Cyclone Center decided on ten classifications per picture to reach a *“statistically reasonable consensus”* [22], the project Pattern Perception used a *“retirement limit”* of 20 votes, in OpenStreetMap 15 contributors per square kilometre resulted in a very good positional accuracy [20] and in the MalariaSpot game 22 votes from non-expert players or 13 votes from trained players resulted in an accuracy higher than 99% [57]. StallCatchers tried to reduce this number through individually weighed sensitivity measures in order to quickly advance the study field and to ensure that the time of the citizens is spent efficiently [58,59]. StallCatchers ultimately arrived at a flexible number of necessary votes per picture based on voting consistency and user experience, with an average of seven votes [58,59]. Such methods could in the future also be implemented in the CrowdWater game, to keep the number of necessary votes per observation flexible, e.g. by checking the voting agreement for each observation and by taking the accuracy of the player into account. For example, an observation, where five usually rather accurate players are in full agreement, is likely to already yield the correct mean value, whereas high disagreement for an observation might require more votes to obtain an accurate classification.

### 4.6 What motivates participants to play the CrowdWater game?

The survey results indicate that the majority of the game players enjoy *“helping others”*, the game in general, or are interested in hydrology. The majority (75%) of the players enjoy classifying difficult pictures, even though they might get fewer points, which also suggests that most users like the game for its purpose, rather than for the gamified aspects. This is in contradiction with the statement that they found it frustrating not to get full points, even when they were sure about their vote. Only a minority of players mentioned that they enjoy the competition but one player indicated that s(he) found the gamified aspects distracting. Based on the survey results, we decided to keep the gamified aspects as they are currently implemented.

The survey also showed that there was a large overlap between the game players and app contributors. This was not what we had expected, as we thought that the different approaches would appeal to different people. However, many of the people we could initially reach with news about the CrowdWater game were already interested in the CrowdWater project. Perhaps in the future, when both parts of the project are better known, the two user groups may become more distinct. One advantage of the overlap between the game players and the app users is that 79% of the game players who participated in the survey indicated that playing the game made them more aware of how to place a staff gauge in the app. This is likely because the players see examples of both good and poorly placed staff gauges in the game, which might make them more aware of how they can do it better themselves. This means that the game could be used as initial training before using the app, as some of the reference pictures do not have the correct angle, size or sometimes lack a staff gauge altogether [39].

### 4.7 Further research

The CrowdWater game can improve the accuracy of the water level class data gathered through the CrowdWater app and thereby enhanced the usability of the data, e.g. for hydrological modelling. Future research will have to investigate how to best incorporate the game results into the app. One method could be to automatically update the app data with the values derived from the game. However, this could also put errors into the app data that were not there before. Alternatively, deviations between the app and game values could be flagged, so that an expert can look at these particular data points. The main issue with the second approach is the scalability, as the database may quickly become too large for such an approach. However, super users who have played more than a certain number of rounds of the game may be involved in this as well, as the accuracy of their data was very high and their votes could potentially be weighed more. The original app value could also be taken into account by simply adding it to the game votes as an additional vote, perhaps with additional weight as the app user gets to see the actual location, whereas the game players only see the picture.

Another interesting topic to be investigated in the future, is that of automating the CrowdWater game. Michelucci and Dickinson [60] defined the phrase *“human computation”* as the *“combination of humans and computers to accomplish a task that neither can do alone”*. This is also reflected in Kawrykow et al. [35] who say that *“crowdsourcing begins where automation fails”*. The boundaries of what a computer can accomplish are however likely to shift in the future, which means that it is feasible that some of the steps that are currently done by game players, such as an automatic recognition of the virtual staff gauge and water level by a computer, could be outsourced to computers. At that stage it might still be possible to continue the game, but to adjust the specific tasks to what is needed. This balance will always have to be reassessed carefully, as *“intuition and reasoning often make humans more effective than computer algorithms in various realms of problem solving*.*”* [21]. Additionally people are better at visual classifications, as shown in projects such as StallCatchers [58,59], Galaxy Zoo [23], Snapshot Serengeti [24] and Cyclone center [22].

## 5. Conclusions

The CrowdWater game allows checking and correcting crowdsourced water level class data based on the pictures that are submitted by citizen scientists through the CrowdWater app. This means that both data submission and data quality control are crowdsourced, which provides two different tasks (one in the field and one online) for citizen scientists who want to join the CrowdWater project. The results of this study indicate that the CrowdWater game improves the accuracy of the water level class data that are collected via the app by correcting a third of all app values. This improves water level class data for future purposes such as hydrological modelling. The game also helped to increase the resolution of the data as a third of all classified pictures had a mean game vote that fell into a half class. This provides higher resolution data than is currently possible through the app. The game can also be used to filter unusable observations or reference pictures through the report function, e.g. if the virtual staff gauge was not placed correctly, but half of the players, including a quarter of the regular players, never used this function.

Through the pictures provided via the app, the game can ensure data quality control for time-series of water level class data obtained via citizen science. We, therefore, recommend that citizen science projects obtain pictures, in addition to an observation value, so that they can be used for data quality control. While other citizen science games so far have mostly used professional pictures, this study shows that games can also be based on crowdsourced pictures of environmental observations. Additionally, we recommend that citizen science games aim for regular contributors through suitable advertisement and achievable daily goals, as regular players tend to have a higher voting accuracy. Games or citizen science projects should also determine early during a project the right numbers of votes per observation, as this has the potential to save time and effort of project organisers and citizen scientists.

Even though the results show that the majority of the submitted crowdsourced water level class data is correct, even without quality control, the results of this study indicate that the CrowdWater game improves the data by correcting water level class observations, increasing the data resolution and removing unusable reference pictures and observations. The results of the CrowdWater game show the potential of gamified approaches to crowdsource data quality control in citizen science projects. This is particularly valuable for variables that can change rapidly, such as water levels in our case, because other forms of data quality control are difficult because observations by different citizen scientists at the same time and place are not realistic in practice.

## Supporting information

S1 File Questions for the online survey for CrowdWater game players. (DOCX)
