# Supplementary material for: The CrowdWater game: A playful way to improve the accuracy of crowdsourced water level class data
Source: PLoS One. 2019 Sep 26;14(9):e0222579. doi: 10.1371/journal.pone.0222579 (PMC6763123; doi:10.1371/journal.pone.0222579)
Supplement: S1 File — (DOCX) [file pone.0222579.s001.docx]

# Supplementary material

## Survey questions

Consent form

- Yes, I give my consent and would like to start the survey.
- No, I do not give my consent and would like to exit the survey.

Have you participated in the CrowdWater game in the past?

- Yes
- No

When did you start playing the game?

- May 2018
- June 2018
- July 2018
- August 2018
- September 2018
- October 2018
- November 2018
- I don’t remember

How often do you (on average) play the CrowdWater game?

- Every day
- 4-6 days per week
- 1-3 days per week
- 2-3 times per month
- Once per month
- I played once

How did you hear about the CrowdWater game?

- A friend, family member or colleague sent me the link
- Via the CrowdWater.ch homepage
- Via the CrowdWater newsletter
- Via the CrowdWater app
- Via the CrowdWater social media channels
- Other…

Did you read the CrowdWater game manual on the homepage?

- Yes
- No

If yes, did you do this before playing the game for the first time or later?

- Before
- Later

Did you watch the CrowdWater game tutorial video?

- Yes
- No

If yes, did you do this before playing the game for the first time or later?

- Before
- Later

What motivates you to play the game?

- I enjoy playing the game.
  - Strongly disagree
  - Disagree
  - Neutral
  - Agree
  - Strongly agree
- I enjoy seeing my own spot update during the game.
  - Strongly disagree
  - Disagree
  - Neutral
  - Agree
  - Strongly agree
- I enjoy seeing how much water levels can change.
  - Strongly disagree
  - Disagree
  - Neutral
  - Agree
  - Strongly agree
- I enjoy helping others.
  - Strongly disagree
  - Disagree
  - Neutral
  - Agree
  - Strongly agree
- I enjoy playing the game more than other citizen science activities because it is very little effort and I don’t have to leave the house.
  - Strongly disagree
  - Disagree
  - Neutral
  - Agree
  - Strongly agree
- I enjoy contributing to science.
  - Strongly disagree
  - Disagree
  - Neutral
  - Agree
  - Strongly agree
- I am interested in hydrology.
  - Strongly disagree
  - Disagree
  - Neutral
  - Agree
  - Strongly agree
- I enjoy competing with others.
  - Strongly disagree
  - Disagree
  - Neutral
  - Agree
  - Strongly agree
- I enjoy the chance to win the competition of the day.
  - Strongly disagree
  - Disagree
  - Neutral
  - Agree
  - Strongly agree
- I enjoy the chance to win the monthly championship.
  - Strongly disagree
  - Disagree
  - Neutral
  - Agree
  - Strongly agree
- I enjoy the challenge to do better than last time.
  - Strongly disagree
  - Disagree
  - Neutral
  - Agree
  - Strongly agree
- I enjoy being part of the CrowdWater community.
  - Strongly disagree
  - Disagree
  - Neutral
  - Agree
  - Strongly agree
- I enjoy helping the environment.
  - Strongly disagree
  - Disagree
  - Neutral
  - Agree
  - Strongly agree
- Other reason:

What aspects do you like most about the game? (Please select all that apply)

- Looking at pictures of rivers
- Seeing my own pictures
- Classifying difficult pictures, even though I might not get full points
- Classifying easy pictures, because I will likely get full points
- Reporting spots (skipping a spot and reporting some problem associated with it, instead of voting for a water level class
- Getting points and prices as a reward for good answers and frequent contributions
- Competing against others
- Other…

Which aspects of the game do you find frustrating? (Please select all that apply.)

- Pictures that are not taken from the same angle
- Water levels that are difficult to classify
- Vegetation that makes it difficult to determine the right level
- Difficulty finding adequate references in the pictures
- Not getting full points, even though I am sure of my vote
- The points and competition are unnecessary and distract from the scientific goal
- I don’t find any aspects frustrating
- Other…

What do you think of the current number of pictures per round (12)?

- There are too many pictures to compare
- This is the right number of pictures to compare
- I wish that there would be more pictures to compare

How long does it take you to finish one round (=12 pictures) on average?

- Less than 5 minutes
- 5-10 minutes
- 10-15 minutes
- 15-20 minutes
- More than 20 minutes

Have you used the CrowdWater app?

- Yes
- No

Conditional forwarding – if participant has used the CrowdWater app:

- Which of the two did you do first, using the app or playing the game?
  - Game
  - App
  - I don’t remember
- Which of the activities do you prefer any why?
  - Game
  - App
  - I enjoy both activities equally
  - Reason:
- Did playing the game help you to be more aware of how to place a staff gauge in the app?
  - No change
  - Small improvement
  - Strong improvement
- Did playing the game help you to estimate water level classes in the app?
  - No change
  - Small improvement
  - Strong improvement
- Has playing the game motivated you to use the app more?
  - Strongly disagree
  - Disagree
  - Neutral
  - Agree
  - Strongly agree
- Do you enjoy the complementarity of collecting data in the app and checking the data in the game?
  - Yes
  - No
  - Neutral
- What motivates you to use the app? (Please select all that apply.)
  - I enjoy seeing how much the water level can change in response to different weather conditions.
  - I enjoy visiting the same river on many occasions and have “adopted” my particular location.
  - I enjoy nice weather and looking at rivers.
  - It is fun to see my updates in the app.
  - It is fun to see other people’s updates.
  - I enjoy being in nature.
  - I enjoy helping others.
  - I enjoy contributing to science.
  - I am interested in hydrology.
  - I enjoy competing with others.
  - I enjoy being part of the CrowdWater community.
  - I enjoy helping the environment.
  - Other…
- Additional comments:

Conditional forwarding – if participant has not used the CrowdWater app:

- Have you thought about downloading and using the app after playing the game?
  - No, I am not interested in using the app.
  - No, I don’t have a compatible smartphone to use the app.
  - After playing the game I have thought about using the app, but have not had the time yet.
  - After playing the game I have downloaded the app but never used it.
- Additional comments:

Demographics

- Age
  - <= 20
  - 21-40
  - 41-60
  - 61-80
  - > 80
- Gender
  - Male
  - Female
  - Other / I prefer not to say
- Highest level of education completed
  - Primary school
  - Secondary school / High school
  - Vocational training (Deutsch: Berufslehre)
  - University / College / Applied University
  - Masters
  - Other…
- What is your country of residence?
  - Switzerland
  - Austria
  - Germany
  - Other…
- Can we link the answers from this survey to your CrowdWater game statistics? If yes, please provide your username or email address below to give us permission to do so, otherwise just leave this question empty. Please note that this is entirely voluntary, you are in no way obliged to give us this permission.

Feedback & comments: If you would like to provide some feedback regarding the survey or have any suggestions of hoe to improve the CrowdWater game please enter them here.
